# Maternal and Neonatal Outcomes in Pregnancies with PCR-Confirmed SARS-CoV-2 Infection Ending in Live Birth: A Single-Centre Turkish Cohort Benchmarked Against National Reference Data

**DOI:** 10.3390/jcm15187069

**Published:** 2026-09-11

**Authors:** Oğuzhan Elçi, Ali Benian

**Affiliations:** 1 Department of Obstetrics and Gynecology, Cerrahpaşa Faculty of Medicine, Istanbul University-Cerrahpaşa, 34098 Istanbul, Türkiye; abenian@iuc.edu.tr; 2 Department of Obstetrics and Gynecology, Başakşehir Çam and Sakura City Hospital, 34480 Istanbul, Türkiye

**Keywords:** COVID-19, SARS-CoV-2, pregnancy, maternal morbidity, neonatal intensive care, COVID-19 vaccines, Türkiye

## Abstract

**Background/Objectives:** Most evidence on SARS-CoV-2 infection in pregnancy comes from hospitalised or symptomatic cohorts sampled early in the pandemic. We described maternal and neonatal outcomes in an unselected cohort of PCR-confirmed infections ending in live birth, identified characteristics associated with neonatal intensive care unit (NICU) admission and with prolonged maternal hospitalisation, and benchmarked event rates against national data. **Methods:** A retrospective cohort of all pregnant women with a positive nasopharyngeal SARS-CoV-2 RT-PCR result at a Turkish tertiary centre between March 2020 and May 2023. Of 151 women identified, 133 had retrievable records; eight pregnancies did not end in a live birth, leaving 125. Prespecified subgroups (NICU admission; maternal stay > 3 days) were compared using Mann–Whitney U and χ^2^/Fisher exact tests, and cohort proportions with 2024 Turkish national rates by one-sample exact binomial tests. **Results:** The mean maternal age was 29.9 ± 5.5 years and gestational age at delivery was 38.0 ± 2.0 weeks; 19 women (15.2%) delivered preterm and 76 (60.8%) were diagnosed in the third trimester. Sixteen neonates (12.8%) required NICU care and two mothers (1.6%) intensive care; there were no maternal deaths or stillbirths. NICU admission was associated with preterm birth (56.2% vs. 9.2%, *p* < 0.001) and lower Apgar scores, and prolonged maternal stay with preterm birth, pregnancy-related comorbidity, and caesarean delivery (all *p* ≤ 0.001). Neither the trimester of infection nor vaccination status was associated with either outcome. Caesarean (64.0%) and preterm birth (15.2%) rates did not exceed national values (*p* = 0.58 and *p* = 0.42). **Conclusions:** Adverse outcomes clustered with preterm delivery and pre-existing pregnancy complications rather than with the timing of infection. Without a matched control group, and with power limited to large effects, these descriptive findings cannot exclude a clinically important effect of SARS-CoV-2.

## 1. Introduction

COVID-19 shaped obstetric practice for three years. By 5 May 2023, when the World Health Organization declared that SARS-CoV-2 no longer constituted a public health emergency of international concern, some 700 million infections and close to seven million deaths had been recorded worldwide. Pregnant women drew attention from the outset. The physiological adaptations of pregnancy (a reduced functional residual capacity, a shift towards a hypercoagulable state, and altered cell-mediated immunity) were expected to worsen the course of a respiratory viral illness. Earlier experience with influenza A(H1N1) and with the SARS-CoV and MERS-CoV epidemics had suggested exactly that [1].

Evidence accumulated quickly and much of it was unfavourable. Pooled analyses linked SARS-CoV-2 infection in pregnancy with preterm birth, preeclampsia, stillbirth, and admission to a neonatal unit [2], and prospective multinational and national datasets confirmed an excess of severe maternal morbidity and mortality from obstetric complications among infected women [3,4]. Two caveats temper this picture. First, a large share of the early literature was drawn from hospitalised or symptomatic cases and from the first two pandemic years. It describes a more severely affected population than the one obstetricians encountered once Omicron became dominant [4]. Second, whether the virus reaches the foetus remains contested: transplacental transmission has been documented convincingly in individual, well-characterised cases [5] and in pooled series [6], while other reviews have been unable to confirm it [7,8].

Vaccination followed a similar arc from uncertainty to reassurance. Pregnant women were excluded from the pivotal trials and recommendations elsewhere were initially cautious. Subsequent systematic reviews of mRNA and viral-vector platforms found no signal of harm in pregnancy alongside evidence of maternal and transplacental antibody responses [9,10,11]. Effectiveness against severe disease has been demonstrated for inactivated vaccines in pregnant populations as well [12]. The Turkish context differs from that of several Western countries in two aspects. The national programme began in January 2021 with the inactivated vaccine CoronaVac and added BNT162b2 in April 2021, so the product available to a pregnant woman depended heavily on when she presented. The Ministry of Health recommended vaccination of pregnant women from the beginning of the programme rather than after a period of exclusion, a recommendation implemented in our own institution, although we were unable to identify a formally published policy document to cite for it.

Reports from unselected obstetric populations spanning the whole pandemic, and from middle-income settings in which an inactivated vaccine predominated early, remain comparatively scarce. We therefore reviewed every pregnancy complicated by a PCR-confirmed SARS-CoV-2 infection managed at our tertiary referral unit between March 2020 and May 2023. Our aims were to describe maternal and neonatal outcomes in the pregnancies that ended in live birth, to examine which characteristics were associated with neonatal intensive care admission and with prolonged maternal hospitalisation, to document the change in vaccination coverage before and after infection, and to place the cohort’s event rates alongside national reference data for Türkiye.

## 2. Materials and Methods

### 2.1. Study Design and Setting

This was a retrospective observational cohort study carried out in the Department of Obstetrics and Gynecology of Istanbul University-Cerrahpaşa, Cerrahpaşa Faculty of Medicine, a tertiary referral and teaching hospital serving the historic peninsula of Istanbul and receiving referrals from across the metropolitan area. Reporting follows the STROBE recommendations for cohort studies [13]; the completed checklist is provided as Table S1.

### 2.2. Participants and Testing

All pregnant women with at least one positive SARS-CoV-2 RT-PCR result recorded during pregnancy between March 2020 and May 2023 were identified from the hospital information system (ISHOP). From March 2020 onwards, a nasopharyngeal swab RT-PCR was performed at our institution on every patient admitted to hospital and on every patient with symptoms compatible with COVID-19. This policy remained in force, applied as clinically indicated, throughout the study period. The ethically approved study protocol explicitly specifies 31 May 2023 as the date for data inclusion in this study. Because admission for delivery triggered testing, a substantial proportion of infections in this cohort were detected at the point of delivery rather than through community or antenatal surveillance.

Symptom status was not recorded prospectively in a structured field, and formal severity grading (oxygen requirement, radiological findings, or WHO clinical progression score) was not available. Three indirect markers of severity could be retrieved from the clinical record and are reported in Section 3.3: receipt of antiviral or antiretroviral therapy, delivery undertaken for COVID-19-related maternal deterioration, and maternal intensive care admission. These are crude proxies and are presented as such; the absence of systematic severity grading is a principal limitation of the study and is discussed in Section 4.1.

Diagnostic testing in Türkiye during this period was qualitative—a specimen was reported as positive or negative—and viral genotyping could not be performed with the methods then in routine use. No variant data were recorded for any patient, and no comparison between variant periods was therefore possible.

**Figure 1:**
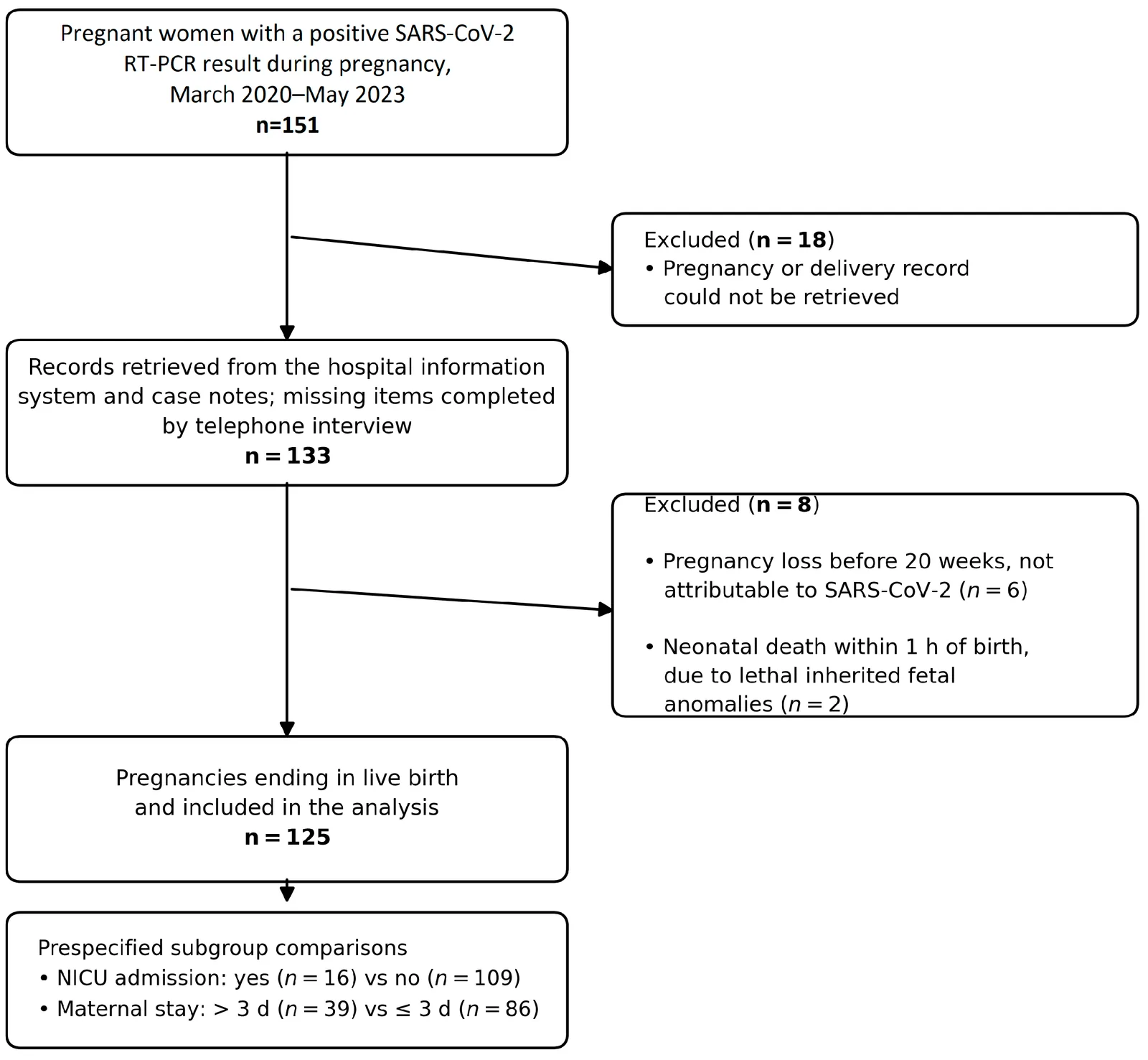
Flow of participants through the study. Of 151 pregnant women with a positive SARS-CoV-2 RT-PCR result identified between March 2020 and May 2023, 18 were excluded because their records could not be retrieved. Of the remaining 133, six pregnancies ended in loss before 20 weeks with no identifiable link to SARS-CoV-2 infection, and two neonates died within the first hour of life from lethal inherited foetal anomalies incompatible with extrauterine survival. One hundred and twenty-five pregnancies ending in live birth were analysed. NICU, neonatal intensive care unit.

The study was restricted by design to pregnancies ending in live birth. Of 151 women identified, 18 were excluded because the pregnancy or delivery record could not be retrieved. Of the remaining 133, a further eight were excluded because the pregnancy did not end in a live birth: six pregnancy losses occurring before 20 weeks, at gestational ages of 7, 10, 11, 12, 16, and 18 weeks, none of which had an identifiable temporal or clinical link to the SARS-CoV-2 infection; and two neonatal deaths within the first hour of life, at 27 and 39 weeks, both attributable to lethal inherited foetal anomalies incompatible with extrauterine survival. One hundred and twenty-five pregnancies remained for analysis (Figure 1). Three of these were twin pregnancies, so 128 neonates were delivered. Denominators therefore differ by level of analysis: pregnancy-level outcomes use *n* = 125, neonate-level counts refer to 128 neonates, and the early neonatal death rate refers to all 127 live births among the 133 pregnancies with retrievable records (Section 3.6).

The rationale for this restriction is that the study’s principal neonatal endpoints—Apgar score and neonatal intensive care admission—are undefined in a pregnancy that does not end in a live birth, so including such pregnancies in the primary analysis would create structurally missing outcome data. Restricting the analysis to live births nevertheless removes the most severe perinatal outcomes from the denominator, and no inference about perinatal mortality attributable to SARS-CoV-2 can be drawn from the primary analysis. To quantify the effect of this decision, a prespecified sensitivity analysis including all 133 pregnancies with retrievable records was performed and is reported in Section 3.7. The eight excluded pregnancies are described explicitly above, with their gestational ages and causes, and individually in Table S2e, so that readers can judge their contribution.

### 2.3. Variables and Definitions

The following were recorded for every woman: age, gravidity, parity, number of previous abortions, gestational age at the first positive PCR result and the corresponding trimester, pre-existing chronic conditions, complications arising during the current pregnancy, COVID-19 vaccines received before and after the infection (product and number of doses), any pharmacological treatment given for COVID-19, mode of delivery, indication for caesarean section, intraoperative complications, maternal and neonatal intensive care admission and length of stay, maternal length of hospital stay, gestational age at delivery, and Apgar scores at one and five minutes. All variables were extracted from the hospital information system and paper case notes. Vaccination status was taken in the first instance from the national immunisation record held within the hospital information system; where a record was incomplete, the woman was contacted by telephone, and the product and number of doses were completed from her recall. Vaccination is therefore the one variable for which a minority of entries rest on patient recall rather than on a documented source, and this is noted as a limitation in Section 4.1.

Trimesters were defined as up to 13 weeks and 6 days, 14 weeks and 0 days to 27 weeks and 6 days, and 28 weeks and 0 days onwards. Preterm birth was defined as delivery before 37 completed weeks. A condition was classified as a chronic medical condition if it predated the current pregnancy and as a complication of the current pregnancy if it was first diagnosed during it. Hypothyroidism, which occurs in both categories in this cohort, was assigned on this basis, so that the two categories are mutually exclusive. For the three twin pregnancies, the lower of the two Apgar scores was used in the per-pregnancy analysis, and a pregnancy was counted as requiring neonatal intensive care if either twin was admitted.

Before the pandemic, the routine post-caesarean stay at our institution was 48 h. During the pandemic, this was extended by institutional decision to 72 h, to allow closer observation of mother and infant. Prolonged maternal hospitalisation was therefore defined a priori as a stay of more than three days. Severe maternal morbidity was considered present if a woman required intensive care admission or transfusion of four or more units of packed red cells, following the American College of Obstetricians and Gynecologists screening criteria [14].

### 2.4. Statistical Analysis

Continuous variables are summarised as the mean ± standard deviation and as the median with interquartile range; categorical variables as counts with percentages. The distribution of continuous variables was examined with the Kolmogorov–Smirnov test, and because several were non-normally distributed, all continuous between-group comparisons used the Mann–Whitney U test. Categorical variables were compared with the χ^2^ test, or with Fisher’s exact test where any expected cell count fell below five. All tests were two-sided, and a *p* < 0.05 was taken as statistically significant. Statistical analyses were performed in Python 3.11 (https://www.python.org) using the SciPy 1.11 (https://scipy.org) and statsmodels 0.14 (https://www.statsmodels.org) libraries. The descriptive statistics and univariable group comparisons were cross-checked against the output of IBM SPSS Statistics version 27.0 (IBM Corp., Armonk, NY, USA) generated for the original thesis analysis; the effect sizes, bootstrap confidence intervals, and multivariable models were computed in Python only. The analysis code is provided as File S3 so that every value reported here can be reproduced from the source data. Effect sizes are reported alongside *p* values: risk differences and odds ratios with 95% confidence intervals for binary variables, and Hodges–Lehmann median differences with bootstrap 95% confidence intervals for continuous variables (Tables S4 and S5).

Several features of the analysis plan should be stated explicitly. No formal sample size calculation was performed; the cohort comprises every eligible woman identified during the study period, and the study is therefore powered only to detect large effects. Approximately fifteen comparisons are made in each of the two subgroup analyses and no adjustment was made for multiple comparisons, so the individual *p* values in those tables should be read as descriptive rather than confirmatory, and effect sizes with confidence intervals should be preferred to a dichotomous reading of statistical significance. Two further analyses were performed at the request of the reviewers and are reported as exploratory. First, a sensitivity analysis repeating the principal comparisons on all 133 pregnancies with retrievable records, including those that did not end in a live birth (Section 3.7, Table S6). Second, an exploratory multivariable logistic regression for each of the two subgroup outcomes, entering preterm birth, complication of the current pregnancy, and maternal age (Section 3.7, Table S7). With 16 neonatal intensive care admissions, the number of events per variable is well below conventional requirements for a stable model, so the multivariable estimates are reported for transparency and should not be treated as adjusted risk estimates.

### 2.5. Comparison with National Reference Data

The study did not include a concurrently recruited group of uninfected pregnant women. To provide an external frame of reference, the proportions observed in the cohort were compared with national figures for Türkiye for 2024, taken from the Health Statistics Yearbook of the Ministry of Health and from Turkish Statistical Institute birth statistics [15,16]. The indicators used were the caesarean share of live births, the preterm birth rate, the maternal mortality ratio, and the neonatal mortality rate. The preterm birth rate was derived by dividing the number of preterm births reported nationally for 2024 by the number of live births reported for that year [16,17].

Each cohort proportion was compared with the corresponding national proportion by a one-sample exact binomial test, with Clopper–Pearson 95% confidence intervals for the cohort estimate. This is an external, ecological comparison and not a matched control group: the reference population is unmatched at the individual level, is drawn from all sectors and regions of the country, and relates to a calendar year that falls after the study period. Its limitations are set out in the Discussion, and the comparison is presented as a benchmark rather than as an estimate of risk attributable to infection.

## 3. Results

### 3.1. Cohort Characteristics

One hundred and twenty-five women were analysed. Their characteristics are shown in Table 1. The mean age was 29.9 ± 5.5 years (median 29, range 21–44) and the median gravidity was 2 (range 1–10); 78 women (62.4%) were parous. Mean gestational age at delivery was 38.0 ± 2.0 weeks (median 38, range 28–40), and 19 women (15.2%) delivered before 37 completed weeks. Mean Apgar scores were 7.4 ± 1.4 at one minute and 8.7 ± 1.1 at five minutes.

The first positive PCR result occurred at a mean gestational age of 27.8 ± 10.6 weeks (median 31). Infection was detected in the third trimester in 76 women (60.8%), in the second in 31 (24.8%), and in the first in 18 (14.4%). Thirty-seven women (29.6%) had a chronic medical condition predating the pregnancy, most often thyroid disease (10 women, 8.0%). Conditions with major organ involvement were uncommon; the most substantial were one woman with systemic lupus erythematosus, Sjögren syndrome, and immune thrombocytopenia, one with HIV infection, and one with restrictive lung disease. Forty-two women (33.6%) developed a complication during pregnancy, most frequently gestational diabetes (16 women, 12.8%) and hypothyroidism first diagnosed in pregnancy (15 women, 12.0%). The diagnosis-level list is given in Table S2.

### 3.2. Vaccination

Thirteen women (10.4%) had received at least one dose of a COVID-19 vaccine before their infection. Ten had received BNT162b2 and three CoronaVac, in one, two, or three doses. After the infection, 59 women (47.2%) had a documented vaccination, most commonly two doses of BNT162b2 (*n* = 28); four women had received both products. The full distribution is given in Table 1. This near five-fold increase is best interpreted as a reflection of vaccine availability across the study period rather than a behavioural response to infection, a point taken up in the Discussion.

### 3.3. Treatment, Delivery, and Outcomes

Forty-five women (36.0%) received pharmacological treatment directed at COVID-19 (Table 2). In 42 of these (93.3% of treated women, 33.6% of the cohort), this consisted of low-molecular-weight heparin alone; one woman received low-molecular-weight heparin with favipiravir, one favipiravir alone, and one lopinavir–ritonavir. No woman received a monoclonal antibody, remdesivir, or systemic corticosteroids as treatment for COVID-19. The effect of corticosteroids on the course of COVID-19 in pregnancy was not established at the time, and departmental policy was to avoid them so as not to risk clinical deterioration or the need for maternal intensive care. Women delivering between 24 and 34 weeks of gestation received a routine two-dose course of betamethasone for foetal lung maturation. Because this is a standard component of care for anticipated preterm birth and was applied uniformly, antenatal corticosteroid exposure was not treated as a study variable and was not analysed separately.

Eighty women (64.0%) delivered by caesarean section. The most common indication was a previous caesarean (38 of 80, 47.5%), followed by cephalopelvic disproportion (13, 16.3%); placenta praevia, breech presentation, maternal disease, failure to progress, and foetal distress each accounted for four cases (5.0%). In one woman, a caesarean section was performed because of deterioration in her general condition attributed to COVID-19. Intraoperative or immediate postpartum complications occurred in five women (4.0%): two uterine artery ligations, two peripartum hysterectomies, and one repair of a bowel serosal injury.

Mean maternal hospital stay was 3.7 ± 2.3 days (median 3, range 1–15), and 39 women (31.2%) stayed longer than three days. Two women (1.6%) required maternal intensive care, for eight and twelve days. Sixteen neonates (12.8%) were admitted to the neonatal intensive care unit, with a mean stay of 15.1 ± 15.5 days (median 10, range 3–60). There were no maternal deaths and no stillbirths among the 125 analysed pregnancies.

The three available proxies for infection severity were as follows. Three women (2.4%) received an antiviral or antiretroviral agent (favipiravir alone in one, favipiravir with low-molecular-weight heparin in one, and lopinavir–ritonavir in one); one woman (0.8%) delivered by caesarean section for deterioration in her general condition attributed to COVID-19; and two women (1.6%) required maternal intensive care. On these markers, severe maternal COVID-19 was uncommon in this cohort, which is consistent with a case mix dominated by mild or incidentally detected infection. These proxies do not substitute for symptom-level severity grading, and the interpretive limits this imposes are set out in Section 4.1.

### 3.4. Neonatal Intensive Care Admission

Neonates admitted to the NICU were delivered significantly earlier than those who were not (35.6 ± 3.7 vs. 38.4 ± 1.3 weeks, *p* = 0.002); 56.2% versus 9.2% were born preterm (risk difference +47.1%, 95% CI +22.2 to +72.0; odds ratio 12.73, 95% CI 3.90–41.53; *p* < 0.001). Apgar scores were lower at one minute (6.3 ± 2.0 vs. 7.5 ± 1.2, *p* = 0.007) and at five minutes (7.8 ± 1.9 vs. 8.8 ± 0.8, *p* = 0.019). Maternal age, gravidity, parity, previous abortions, the gestational age at which infection was detected, and the trimester of infection did not differ between the groups (Table 3).

Vaccination before or after infection was not associated with NICU admission. Women whose neonates required intensive care were more likely to have received treatment for COVID-19 (68.8% vs. 31.2%; risk difference +37.6%, 95% CI +13.2 to +61.9; odds ratio 4.85, 95% CI 1.56–15.06; *p* = 0.003). This association is confounded by indication: low-molecular-weight heparin was prescribed as venous thromboembolism prophylaxis to women whose admission was prolonged, and prolonged admission in turn accompanied preterm and complicated deliveries. It should not be read as an effect of treatment. Chronic conditions (18.8% vs. 31.2%, *p* = 0.390), pregnancy complications (50.0% vs. 31.2%, *p* = 0.137), mode of delivery (81.2% vs. 61.5% caesarean, *p* = 0.124), intraoperative complications (6.2% vs. 3.7%, *p* = 0.502), maternal length of stay (50.0% vs. 28.4% beyond three days, *p* = 0.092), and maternal intensive care admission (0% vs. 1.8%, *p* = 1.000) did not differ significantly between the groups. Effect sizes with confidence intervals for every comparison are given in Table S4.

### 3.5. Prolonged Maternal Hospitalisation

Women who stayed longer than three days delivered earlier (37.1 ± 2.7 vs. 38.5 ± 1.4 weeks, *p* = 0.001), more often delivered preterm (30.8% vs. 8.1%; risk difference +22.6%, 95% CI +7.0 to +38.2; odds ratio 5.02, 95% CI 1.79–14.04; *p* = 0.001), and their neonates had lower Apgar scores at one and five minutes (both *p* < 0.001). They were more likely to have developed a complication during pregnancy (56.4% vs. 23.3%; risk difference +33.2%, 95% CI +15.2 to +51.1; odds ratio 4.27, 95% CI 1.91–9.57; *p* < 0.001) and to have delivered by caesarean section (92.3% vs. 51.2%; risk difference +41.1%, 95% CI +27.7 to +54.6; odds ratio 11.45, 95% CI 3.28–40.04; *p* < 0.001), and more had received treatment for COVID-19 (59.0% vs. 25.6%, *p* < 0.001)—again, largely thromboprophylaxis given because of the admission itself (Table 4).

Maternal age, gravidity, parity, previous abortions, chronic conditions, the gestational age and trimester at which infection was detected, vaccination before or after infection, intraoperative complications, maternal intensive care admission, and NICU admission did not differ significantly between the two groups, although vaccination after infection (59.0% vs. 41.9%, *p* = 0.076), maternal intensive care (5.1% vs. 0%, *p* = 0.096), and NICU admission (20.5% vs. 9.3%, *p* = 0.092) did not reach conventional significance. We draw no inference from these three comparisons: each has a confidence interval that includes no difference (for example, NICU admission risk difference +11.2%, 95% CI −2.9 to +25.3), and with fifteen unadjusted comparisons in this table, a *p* value between 0.05 and 0.10 carries little evidential weight. Effect sizes for every comparison are given in Table S5.

### 3.6. Cohort Event Rates Against National Reference Data

Cohort event rates set against 2024 national figures for Türkiye are shown in Table 5. The caesarean rate of 64.0% (95% CI 54.9–72.4) did not differ significantly from the national caesarean share of live births of 61.2% (*p* = 0.58). The preterm birth rate of 15.2% (95% CI 9.4–22.7) did not differ significantly from the national rate of 12.9% (*p* = 0.42). There were no maternal deaths, against a national maternal mortality ratio of 11.5 per 100,000 live births, and no stillbirths. Among the 127 live births in the wider identified cohort, the two early neonatal deaths—both attributable to lethal inherited anomalies—gave a rate of 1.6% (95% CI 0.2–5.6), which did not differ significantly from the national neonatal mortality rate of 5.6 per 1000 (*p* = 0.16). Because national figures for a given indicator vary slightly between reporting years and sources, we repeated the two comparisons of substantive interest across the plausible range of published values. The caesarean comparison remained non-significant for any national value between 58% and 63% (*p* ≥ 0.20), and the preterm comparison for any value between 11.0% and 13.5% (*p* ≥ 0.15); neither conclusion therefore depends on the precise reference figure adopted.

No national statistic is published for neonatal intensive care admission or for maternal length of hospital stay, so the two outcomes used in the subgroup analyses could not be benchmarked externally.

### 3.7. Sensitivity and Exploratory Multivariable Analyses

Repeating the principal comparisons on all 133 pregnancies with retrievable records did not change any conclusion (Table S6). Among the 127 births at or beyond 20 weeks, the preterm birth rate was 15.7% (95% CI 9.9–23.3) against the national rate of 12.9% (*p* = 0.35), and the caesarean rate was 63.8% (95% CI 54.8–72.1) against 61.2% (*p* = 0.59). The associations between preterm birth and neonatal intensive care admission (*p* < 0.001) and between preterm birth and prolonged maternal stay (*p* = 0.036) persisted. Counting all eight non-live-birth outcomes as adverse events, the composite of neonatal intensive care admission, maternal intensive care admission, or a pregnancy not ending in live birth occurred in 26 of 133 pregnancies (19.5%).

In exploratory multivariable logistic regression, preterm birth remained associated with neonatal intensive care admission after adjustment for pregnancy complication and maternal age (adjusted odds ratio 14.20, 95% CI 3.60–56.05), and both preterm birth (adjusted odds ratio 3.09, 95% CI 1.01–9.41) and complication of the current pregnancy (adjusted odds ratio 3.16, 95% CI 1.34–7.49) remained associated with prolonged maternal stay (Table S7). With 16 and 39 events respectively, these models are underpowered and their confidence intervals are wide; they are reported to demonstrate that the univariable associations are not obviously explained by the covariates entered, and not as adjusted risk estimates.

## 4. Discussion

In 125 pregnancies complicated by PCR-confirmed SARS-CoV-2 infection and ending in live birth, we found no maternal deaths, two maternal intensive care admissions, and a NICU admission rate of 12.8%. The characteristics that separated women and neonates with adverse outcomes from the rest were obstetric rather than infectious: preterm delivery, pre-existing complications of the pregnancy, and caesarean delivery. Neither the trimester in which infection was detected nor vaccination status was associated with NICU admission or prolonged maternal stay, and the cohort’s caesarean, preterm birth, maternal mortality, and stillbirth rates did not exceed national values.

These findings sit at the reassuring end of a literature whose central tendency is less reassuring. Large multinational and registry-based studies have shown clear excesses of severe maternal morbidity, intensive care admission, and mortality among infected pregnant women [3,4,18], and pooled obstetric outcome data have shown increases in preterm birth, preeclampsia, and neonatal unit admission [2]. The discrepancy is best explained by case mix. Studies reporting large effects have generally sampled hospitalised or symptomatic women, whereas our cohort was assembled from PCR results under a universal admission-testing policy and therefore includes many women whose infection was detected incidentally, on admission in labour. Other unselected cohorts have likewise failed to demonstrate an association between infection and adverse obstetric outcome [19,20]. Ours should be read as a description of what an obstetric service sees across a whole pandemic, not as a test of the virus’s pathogenic potential in severe disease.

The predominance of third-trimester detection (60.8%) deserves emphasis for the same reason. Antenatal attendance fell sharply during the pandemic in Türkiye as elsewhere, and our own antenatal clinic suspended elective appointments for part of the study period, while testing was applied to every hospital admission; many women were therefore first tested when they presented to deliver. First- and second-trimester infections were almost certainly under-ascertained, and any inference about the timing of infection—including our own null finding—is weakened accordingly. That surveillance artefact is itself a health-service finding worth stating: a pandemic response that interrupts routine antenatal care displaces the detection of infection to the point of delivery, when there is no longer any opportunity to modify management.

Chronic disease has been a consistent predictor of severe COVID-19 in the general population [21,22]. Just under a third of our cohort (29.6%) carried a chronic diagnosis, but the list was dominated by thyroid disease, migraine, asthma, and heterozygous factor V Leiden; only a handful of women had a disease with substantial organ involvement. A cohort of this composition and size cannot be expected to reproduce the associations reported in populations carrying a heavier burden of cardiometabolic disease.

Two pregnancy complications were common enough in this cohort to deserve separate comment. Gestational diabetes was recorded in 16 women (12.8%) and hypothyroidism first diagnosed in pregnancy in 15 (12.0%). The gestational diabetes rate is unremarkable against published figures, which range from about 7% to over 16% depending on screening strategy and diagnostic threshold, and our estimate is statistically indistinguishable from reference values of 10%, 12.5%, or 16.5% (*p* = 0.30, 0.89, and 0.33 respectively); it differs only from a low reference of 7.5% (*p* = 0.039). No comparable national statistic is published for either condition in Türkiye, so these comparisons are indicative only. Because both conditions independently predict caesarean delivery and longer admission, we examined whether they could account for the associations in Table 3 and Table 4. Neither did: gestational diabetes was not significantly associated with caesarean delivery (75.0% vs. 62.4%, *p* = 0.33), prolonged stay (43.8% vs. 29.4%, *p* = 0.26), or neonatal intensive care admission (12.5% vs. 12.8%, *p* = 1.00), and the same held for pregnancy-onset hypothyroidism. Whether SARS-CoV-2 exerts a direct endocrine or metabolic effect in pregnancy cannot be addressed by a cohort without an uninfected comparison group, and we make no claim in either direction.

Vaccination coverage rose from 10.4% before infection to 47.2% afterwards. It is tempting to read this as behaviour change following illness, but the interpretation does not survive scrutiny: no vaccine was available in Türkiye before January 2021, so the two proportions are separated by calendar time as much as by the infection itself. What the figures do show is how low coverage was among pregnant women during the first year of the programme, at a point when the national recommendation already included them and when vaccination was known to be safe and effective in pregnancy [9,10,11,12]. We could not assess vaccine effectiveness, since by design every woman had been infected and only 13 had been vaccinated beforehand.

The associations we did detect are largely mediated by gestational age. Preterm delivery drives low Apgar scores, NICU admission, and prolonged maternal stay, and it is unsurprising that these variables move together; we report them because they characterise the cohort, not because they identify independent risk factors. The finding that women treated for COVID-19 had worse outcomes is a clear instance of confounding by indication rather than a treatment effect, since low-molecular-weight heparin was given as thromboprophylaxis precisely to women whose stay was prolonged. It supports no inference about anticoagulation, which in COVID-19 remains a question for randomised trials [23].

A further observation extends beyond the COVID-19 question. Independently of what caused them, the adverse pregnancy outcomes recorded in this cohort are now recognised as sex-specific markers of later maternal cardiovascular risk. Nineteen women (15.2%) delivered preterm, 16 (12.8%) developed gestational diabetes, and 4 (3.2%) a hypertensive disorder of pregnancy; 31 women (24.8%) had at least one of these. Pregnancy acts as a cardiovascular stress test, and an unfavourable maternal cardiometabolic phenotype can both precipitate adverse pregnancy outcomes and predict later cardiovascular disease, with effects that extend to the offspring through developmental programming [24]. On current preventive frameworks, which emphasise the postpartum period as a window for cardiovascular risk assessment rather than a purely obstetric endpoint, roughly a quarter of the women in this cohort would meet criteria for structured postpartum cardiovascular counselling and follow-up. That recommendation does not depend on whether SARS-CoV-2 contributed to the outcome, and it is an actionable message for services caring for women after an affected pregnancy.

### 4.1. Strengths and Limitations

There are several strengths of this study. First, every woman was assessed and delivered at a single tertiary centre by the same clinical team. Second, laboratory confirmation was required for study entry, and testing was systematically applied to all hospital admissions, minimising case ascertainment bias. In addition, outcome data were available for all participants until hospital discharge. Finally, the cohort spans the entire pandemic rather than a single wave.

This study is descriptive. It estimates associations within a cohort of infected pregnancies and cannot support causal statements about the effect of SARS-CoV-2, and the language throughout has been chosen with that constraint in mind. The limitations are substantial and shape what can be concluded. The most important is the absence of a concurrently recruited uninfected comparison group. The national benchmark in Table 5 mitigates this only partly, and its weaknesses should be stated plainly: the reference population is unmatched at the individual level, so no adjustment for age, parity, body mass index, or comorbidity is possible; it combines public, university, and private-sector deliveries, and caesarean rates differ markedly between these sectors; and it relates to 2024, a calendar year falling after the study period. One point does run in the study’s favour. A tertiary referral centre concentrates high-risk pregnancies and a high proportion of women with a previous caesarean—47.5% of our caesareans were repeat procedures—so the expected direction of bias is towards higher event rates in our cohort than in the national population; that we did not observe higher rates is therefore modestly reassuring. It is not, however, equivalent to a matched comparison, and the study cannot attribute the outcomes it describes to infection.

Second, the cohort is small. With 16 NICU admissions and two maternal intensive care admissions, the study has adequate power only for large effects, and the absence of statistically significant differences should not be mistaken for evidence of equivalence. The upper 95% confidence limit for maternal mortality in a cohort of 125 with no deaths is 2.9%, far above any plausible effect size; that comparison is reported only for completeness. Third, restricting analysis to live births excluded six pregnancy losses before 20 weeks and two neonatal deaths from lethal inherited anomalies. Neither group is plausibly attributable to SARS-CoV-2 on clinical grounds, but their exclusion still means that no conclusion about perinatal mortality can be drawn from the primary analysis.

Fourth, disease severity was not graded: we have no systematic record of symptoms, oxygen requirement, radiological findings, or WHO clinical progression score, and a cohort in which severity is unmeasured cannot distinguish asymptomatic from critical illness. Fifth, no variant information was available. Genotyping was not performed with the diagnostic methods in routine use in Türkiye during the study period, and no variant data were recorded, so infections could not be assigned to a variant and no comparison between variant periods was undertaken. This matters, because virulence in pregnancy differed substantially between periods: in a population-based Canadian study, the risk of severe maternal morbidity was roughly threefold during the wild-type, Alpha, and Delta periods but only 1.2-fold during Omicron [18]. A cohort spanning all four periods without stratification necessarily produces a diluted, averaged estimate. Sixth, although no analysis variable had missing values in the final dataset, a small number of items were completed by telephone recall rather than from a documented source, which is vulnerable to error; this applies principally to vaccine product and dose. Finally, follow-up ended at discharge, so neither long COVID in the mothers [25] nor the neurodevelopmental outcome of infants exposed in utero could be examined.

### 4.2. Implications

Two practical points follow. The first concerns service design rather than virology: the concentration of diagnoses at the point of delivery reflects a system in which pregnant women lost access to routine antenatal care, and any future pandemic plan should treat continuity of antenatal care as an outcome in its own right. The second concerns the evidence itself. Cohorts of this size will not settle questions about maternal and perinatal risk; what they can contribute is unselected, laboratory-confirmed data from settings under-represented in the international literature, and they are most useful when pooled. We encourage the assembly of national registries with harmonised definitions of severity and variant period, so that data such as ours can be combined rather than read in isolation.

## 5. Conclusions

In an unselected single-centre cohort of 125 pregnancies with PCR-confirmed SARS-CoV-2 infection ending in live birth, there were no maternal deaths and adverse outcomes were uncommon. Neonatal intensive care admission and prolonged maternal hospitalisation were associated with preterm delivery, lower Apgar scores, pregnancy-related comorbidity, and caesarean delivery, but not with the trimester in which infection was detected or with vaccination status, and cohort event rates did not exceed national reference values for Türkiye. Because that national comparison is ecological rather than matched, because pregnancy losses were excluded, and because the study is powered only for large effects, these results describe outcomes in infected pregnancies rather than quantify risk attributable to infection. Larger, controlled, and period-stratified studies, together with the long-term follow-up of exposed infants, remain necessary.

## Figures and Tables

**Table 1:** Demographic and clinical characteristics of the 125 pregnancies with PCR-confirmed SARS-CoV-2 infection ending in live birth.

Characteristic	*n* (%) or Mean ± SD	Median (Range)
Age, years	29.90 ± 5.45	29 (21–44)
Gravidity	2.57 ± 1.65	2 (1–10)
Parous	78 (62.4)	—
Parity, among parous women	1.86 ± 1.24	1 (1–8)
Gestational age at delivery, weeks	38.03 ± 2.00	38 (28–40)
**Preterm birth (<37 weeks)**	**19 (15.2)**	—
Twin pregnancy	3 (2.4)	—
Apgar score at 1 min	7.37 ± 1.38	8 (2–10)
Apgar score at 5 min	8.70 ± 1.06	9 (3–10)
Gestational age at first positive PCR, weeks	27.76 ± 10.61	31 (5–40)
**Trimester of first positive PCR**		
First	18 (14.4)	—
Second	31 (24.8)	—
Third	76 (60.8)	—
**Vaccination before infection**	**13 (10.4)**	—
BNT162b2, 1/2/3 doses	4/4/2	—
CoronaVac, 1/2/3 doses	1/1/1	—
**Vaccination after infection**	**59 (47.2)**	—
BNT162b2, 1/2/3/4 doses	14/28/5/3	—
CoronaVac, 1/2/4 doses	2/2/1	—
Both products	4	—
**Any chronic medical condition**	**37 (29.6)**	—
Thyroid disorder	10 (8.0)	—
Cardiovascular disorder	6 (4.8)	—
Thrombophilia or haematological disorder	6 (4.8)	—
Asthma or chronic respiratory disease	5 (4.0)	—
Neurological disorder	5 (4.0)	—
Autoimmune or autoinflammatory disease	3 (2.4)	—
Varicose veins	2 (1.6)	—
Current or previous malignancy	2 (1.6)	—
Other	6 (4.8)	—
**Any complication of the current pregnancy**	**42 (33.6)**	—
Gestational diabetes	16 (12.8)	—
Hypothyroidism first diagnosed in pregnancy	15 (12.0)	—
Placenta praevia totalis	4 (3.2)	—
Hydronephrosis	4 (3.2)	—
Intrahepatic cholestasis of pregnancy	3 (2.4)	—
Preeclampsia	3 (2.4)	—
Gestational hypertension	1 (0.8)	—
PPROM with chorioamnionitis	1 (0.8)	—

SD, standard deviation; PPROM, preterm prelabour rupture of membranes. Bold text is used for group headings. A diagnosis was classified as a chronic medical condition if it predated the current pregnancy and as a complication of the current pregnancy if it was first made during it; the two categories are therefore mutually exclusive. Subcategories within each group are not mutually exclusive: seven women had more than one chronic diagnosis and five more than one pregnancy complication. Other chronic conditions comprise obesity, HIV infection, neurogenic bladder, achalasia, kyphoscoliosis, and hepatic steatosis. The complete diagnosis-level list is given in Table S2.

**Table 2:** Treatment for COVID-19, mode of delivery, and outcomes (*n* = 125).

Variable	*n* (%)
**Pharmacological treatment for COVID-19**	45 (36.0)
Low-molecular-weight heparin alone	42 (33.6)
Low-molecular-weight heparin + favipiravir	1 (0.8)
Favipiravir alone	1 (0.8)
Lopinavir–ritonavir	1 (0.8)
Systemic corticosteroids as COVID-19 treatment	0 (0)
**Mode of delivery**	
Caesarean section	80 (64.0)
Vaginal delivery	45 (36.0)
**Indication for caesarean section (*n* = 80)**	
Previous caesarean section	38 (47.5)
Cephalopelvic disproportion	13 (16.3)
Placenta praevia totalis	4 (5.0)
Breech presentation	4 (5.0)
Maternal disease	4 (5.0)
Failure to progress in labour	4 (5.0)
Foetal distress	4 (5.0)
Twin pregnancy	3 (3.8)
Placental abruption	2 (2.5)
Severe preeclampsia	2 (2.5)
Chorioamnionitis	1 (1.3)
Foetal anomaly	1 (1.3)
Foetal macrosomia	1 (1.3)
COVID-19 with maternal deterioration	1 (1.3)
**Intraoperative or immediate postpartum complication**	5 (4.0)
Uterine artery ligation	2 (1.6)
Peripartum hysterectomy	2 (1.6)
Repair of bowel serosal injury	1 (0.8)
**Maternal hospital stay > 3 days**	39 (31.2)
Maternal hospital stay, days, mean ± SD (median, range)	3.65 ± 2.34 (3, 1–15)
**Maternal intensive care admission**	2 (1.6)
**Neonatal intensive care admission**	16 (12.8)
Neonatal intensive care stay, days, mean ± SD (median, range)	15.12 ± 15.45 (10, 3–60)
**Stillbirth**	0 (0)
**Maternal death**	0 (0)

SD, standard deviation. Bold text is used for group headings. Percentages for caesarean indications are of the 80 women who delivered by caesarean section; two women had two recorded indications, so the subcategories sum to more than 80. Two women had a previous caesarean together with chorioamnionitis or severe preeclampsia, respectively.

**Table 3:** Comparison of pregnancies by neonatal intensive care unit admission.

Variable	No NICU (*n* = 109)	Median (IQR)	NICU (*n* = 16)	Median (IQR)	*p*
Age, years	29.83 ± 5.53	29 (25–34)	30.44 ± 5.01	30.5 (27.5–32.8)	0.524
Gravidity	2.61 ± 1.73	2 (1–3)	2.31 ± 0.95	2 (2–3)	0.924
Parity	1.18 ± 1.38	1 (0–2)	1.00 ± 0.89	1 (0–1)	0.966
Previous abortions	0.33 ± 0.81	0 (0–0)	0.25 ± 0.45	0 (0–0)	0.940
**Gestational age at delivery, weeks**	**38.39 ± 1.30**	**39 (38–39)**	**35.56 ± 3.65**	**35.5 (34–39)**	**0.002**
**Apgar score at 1 min**	**7.52 ± 1.21**	**8 (7–8)**	**6.31 ± 1.96**	**7 (5–7)**	**0.007**
**Apgar score at 5 min**	**8.83 ± 0.81**	**9 (8–9)**	**7.81 ± 1.91**	**8.5 (7–9)**	**0.019**
Gestational age at positive PCR, weeks	28.23 ± 10.66	32 (20–38)	24.56 ± 9.98	27.5 (18–32)	0.082
Trimester I/II/III, *n*	15/26/68	—	3/5/8	—	0.637 ^b^
**Preterm birth < 37 weeks**	**10 (9.2)**	—	**9 (56.2)**	—	**<0.001 ** ^b^
Vaccinated before infection	11 (10.0)	—	2 (12.5)	—	0.670 ^b^
Vaccinated after infection	50 (45.9)	—	9 (56.2)	—	0.437 ^a^
**Treated for COVID-19**	**34 (31.2)**	—	**11 (68.8)**	—	**0.003 ** ^a^
Chronic medical condition	34 (31.2)	—	3 (18.8)	—	0.390 ^b^
Complication of current pregnancy	34 (31.2)	—	8 (50.0)	—	0.137 ^a^
Caesarean delivery	67 (61.5)	—	13 (81.2)	—	0.124 ^a^
Intraoperative complication	4 (3.7)	—	1 (6.2)	—	0.502 ^b^
Maternal stay > 3 days	31 (28.4)	—	8 (50.0)	—	0.092 ^b^
Maternal intensive care	2 (1.8)	—	0 (0)	—	1.000 ^b^

SD, standard deviation; IQR, interquartile range; NICU, neonatal intensive care unit. Continuous variables were compared with the Mann–Whitney U test; ^a^ χ^2^ test; ^b^ Fisher’s exact test. Significant results are shown in bold. Approximately fifteen comparisons are presented in this table and no adjustment was made for multiple comparisons; the *p* values should therefore be read as descriptive rather than confirmatory, and effect sizes with 95% confidence intervals (Tables S4 and S5) should be preferred to a dichotomous significant-versus-not-significant reading. Continuous variables are summarised as the mean ± SD and as the median with interquartile range.

**Table 4:** Comparison of pregnancies by duration of maternal hospitalisation.

Variable	≤3 Days (*n* = 86)	Median (IQR)	>3 Days (*n* = 39)	Median (IQR)	*p*
Age, years	29.44 ± 5.62	29 (25–34)	30.92 ± 4.97	30 (28–35)	0.117
Gravidity	2.59 ± 1.76	2 (1–3)	2.51 ± 1.39	2 (2–3)	0.854
Parity	1.15 ± 1.35	1 (0–2)	1.18 ± 1.30	1 (0–2)	0.900
Previous abortions	0.35 ± 0.88	0 (0–0)	0.26 ± 0.44	0 (0–1)	0.805
**Gestational age at delivery, weeks**	**38.48 ± 1.40**	**39 (38–40)**	**37.05 ± 2.68**	**38 (36–39)**	**0.001**
**Apgar score at 1 min**	**7.73 ± 1.00**	**8 (7–8)**	**6.56 ± 1.74**	**7 (6–8)**	**<0.001**
**Apgar score at 5 min**	**8.95 ± 0.68**	**9 (9–9)**	**8.13 ± 1.47**	**8 (8–9)**	**<0.001**
Gestational age at positive PCR, weeks	28.33 ± 10.54	32 (20–38)	26.51 ± 10.79	30 (19–36)	0.312
Trimester I/II/III, *n*	11/21/54	—	7/10/22	—	0.708 ^a^
**Preterm birth < 37 weeks**	**7 (8.1)**	—	**12 (30.8)**	—	**0.001 ** ^a^
Vaccinated before infection	10 (11.6)	—	3 (7.7)	—	0.753 ^b^
Vaccinated after infection	36 (41.9)	—	23 (59.0)	—	0.076 ^a^
**Treated for COVID-19**	**22 (25.6)**	—	**23 (59.0)**	—	**<0.001 ** ^a^
Chronic medical condition	24 (27.9)	—	13 (33.3)	—	0.538 ^a^
**Complication of current pregnancy**	**20 (23.3)**	—	**22 (56.4)**	—	**<0.001 ** ^a^
**Caesarean delivery**	**44 (51.2)**	—	**36 (92.3)**	—	**<0.001 ** ^a^
Intraoperative complication	2 (2.3)	—	3 (7.7)	—	0.175 ^b^
Maternal intensive care	0 (0)	—	2 (5.1)	—	0.096 ^b^
NICU admission	8 (9.3)	—	8 (20.5)	—	0.092 ^b^

SD, standard deviation; IQR, interquartile range; NICU, neonatal intensive care unit. Continuous variables were compared with the Mann–Whitney U test; ^a^ χ^2^ test; ^b^ Fisher’s exact test. Significant results are shown in bold. Approximately fifteen comparisons are presented in this table and no adjustment was made for multiple comparisons; the *p* values should therefore be read as descriptive rather than confirmatory, and effect sizes with 95% confidence intervals (Tables S4 and S5) should be preferred to a dichotomous significant-versus-not-significant reading. Continuous variables are summarised as the mean ± SD and as the median with interquartile range.

**Table 5:** Cohort event rates compared with national reference data for Türkiye, 2024.

Outcome	Cohort, *n*/*N* (%)	Cohort 95% CI	National Rate	*p*
Caesarean delivery	80/125 (64.0)	54.9–72.4	61.2%	0.58
Preterm birth < 37 weeks	19/125 (15.2)	9.4–22.7	12.9%	0.42
Stillbirth	0/125 (0)	0–2.9	—	1.00
Maternal death	0/125 (0)	0–2.9	11.5/100,000	1.00
Early neonatal death ^a^	2/127 (1.6)	0.2–5.6	5.6/1000	0.16

CI, confidence interval. Cohort proportions are compared with the national rate by a one-sample exact binomial test; confidence intervals are Clopper–Pearson. National figures are for 2024 and are taken from the Health Statistics Yearbook of the Ministry of Health and from Turkish Statistical Institute birth statistics [15,16,17]; the national preterm birth rate is derived as 121,067 preterm births divided by 937,559 live births. The caesarean comparison is unchanged in sensitivity analysis; it remains non-significant for any national value between 58% and 63%, and the preterm comparison for any value between 11.0% and 13.5%. ^a^ This row deliberately uses a wider denominator than the rest of the table. Early neonatal death is by definition an outcome of live birth, and the two deaths occurred in pregnancies excluded from the analysis cohort precisely because they did not survive the first hour of life. Referring them to the 125-pregnancy cohort would place the events outside their own denominator, so the rate is calculated over all 127 live births among the 133 pregnancies with retrievable records (125 analysed pregnancies plus the two neonatal deaths). This is a deliberate methodological choice and not an inconsistency. Both deaths were attributable to lethal inherited foetal anomalies. The maternal mortality comparison is presented for completeness only: with no deaths in 125 pregnancies, the upper confidence limit is 2.9%, so the test has essentially no power against a national ratio of 11.5 per 100,000.

## Data Availability

The anonymised dataset analysed during the current study is available from the corresponding author upon reasonable request.

## Supplementary Materials

The following supporting information can be downloaded at: https://www.mdpi.com/article/10.3390/jcm15187069/s1, Table S1: STROBE checklist for cohort studies; Table S2: Diagnosis-level data for the 125 analysed pregnancies; File S3: Analysis code, data dictionary, and coding rules; Table S4: Effect sizes for the comparisons in Table 3 (neonatal intensive care unit admission); Table S5: Effect sizes for the comparisons in Table 4 (maternal hospital stay > 3 days); Table S6: Sensitivity analysis including all 133 pregnancies with retrievable records; Table S7: Exploratory multivariable logistic regression. File S8. Elci Analysis data.
