# Supplementary material for: Maternal and Neonatal Outcomes in Pregnancies with PCR-Confirmed SARS-CoV-2 Infection Ending in Live Birth: A Single-Centre Turkish Cohort Benchmarked Against National Reference Data
Source: J Clin Med. 2026 Sep 11;15(18):7069. doi: 10.3390/jcm15187069 (PMC13607355; doi:10.3390/jcm15187069)
Supplement: Supplementary file 1 [file jcm-15-07069-s001.zip › Elci O Supplementary Tables S1 S2.pdf]

Supplementary material

Maternal and neonatal outcomes in pregnancies with PCR-confirmed SARS-CoV-2 infection ending in live birth: a single-centre Turkish cohort benchmarked against national reference data

Supplementary Table S1. STROBE checklist for cohort studies

Item numbers and descriptors follow von Elm et al., Lancet 2007;370:1453–7. The right-hand column indicates where each item is addressed in the manuscript.

Item	Section	Recommendation	Addressed in
1	Title and abstract	(a) Indicate the study's design with a commonly used term in the title or the abstract. (b) Provide in the abstract an informative and balanced summary of what was done and what was found.	Title; Abstract
2	Background / rationale	Explain the scientific background and rationale for the investigation being reported.	Introduction, paras 1–3
3	Objectives	State specific objectives, including any prespecified hypotheses.	Introduction, para 4
4	Study design	Present key elements of study design early in the paper.	Section 2.1
5	Setting	Describe the setting, locations, and relevant dates, including periods of recruitment, exposure, follow-up and data collection.	Section 2.1, 2.2
6	Participants	(a) Give the eligibility criteria, and the sources and methods of selection of participants; describe methods of follow-up. (b) For matched studies, give matching criteria and number of exposed and unexposed.	Section 2.2; Figure 1. (b) not applicable
7	Variables	Clearly define all outcomes, exposures, predictors, potential confounders and effect modifiers; give diagnostic criteria if applicable.	Section 2.3
8	Data sources / measurement	For each variable of interest, give sources of data and details of methods of assessment. Describe comparability of assessment methods if there is more than one group.	Sections 2.2, 2.3
9	Bias	Describe any efforts to address potential sources of bias.	Sections 2.2, 2.5; Discussion 4.1
10	Study size	Explain how the study size was arrived at.	Section 2.4, para 2
11	Quantitative variables	Explain how quantitative variables were handled in the analyses; if applicable, describe which groupings were chosen and why.	Sections 2.3, 2.4
12	Statistical methods	(a) Describe all statistical methods, including those used to control for confounding. (b) Describe any methods used to examine subgroups and interactions. (c) Explain how missing data were addressed. (d) If applicable, explain how loss to follow-up was addressed. (e) Describe any sensitivity analyses.	Sections 2.4, 2.5
13	Participants	(a) Report numbers of individuals at each stage of the study. (b) Give reasons for non-participation at each stage. (c) Consider use of a flow diagram.	Section 2.2; Figure 1; Supplementary Table S1e
14	Descriptive data	(a) Give characteristics of study participants and information on exposures and potential confounders. (b) Indicate number of participants with missing data for each variable of interest. (c) Summarise follow-up time.	Section 3.1; Tables 1 and 2. Follow-up ended at hospital discharge (Section 4.1)
15	Outcome data	Report numbers of outcome events or summary measures over time.	Section 3.3; Table 2

16	Main results	(a) Give unadjusted estimates and, if applicable, confounder-adjusted estimates and their precision. (b) Report category boundaries when continuous variables were categorised. (c) If relevant, consider translating estimates of relative risk into absolute risk.	Sections 3.4–3.6; Tables 3–5. Estimates are unadjusted; no multivariable model was fitted (Section 2.4)
17	Other analyses	Report other analyses done — e.g. analyses of subgroups and interactions, and sensitivity analyses.	Section 3.6; Table 5
18	Key results	Summarise key results with reference to study objectives.	Discussion, para 1; Conclusion
19	Limitations	Discuss limitations of the study, taking into account sources of potential bias or imprecision. Discuss both direction and magnitude of any potential bias.	Section 4.1
20	Interpretation	Give a cautious overall interpretation of results considering objectives, limitations, multiplicity of analyses, results from similar studies, and other relevant evidence.	Discussion, paras 2–6; Conclusion
21	Generalisability	Discuss the generalisability (external validity) of the study results.	Sections 4.1, 4.2
22	Funding	Give the source of funding and the role of the funders for the present study and, if applicable, for the original study on which the present article is based.	Declarations — Funding

Note. Item 6(b) and item 12(d) are not applicable: the study included no matched comparison group and follow-up was complete to hospital discharge for every participant.

Supplementary Table S2. Diagnosis-level data for the 125 analysed pregnancies

Diagnoses are listed exactly as recorded in the clinical record. Combined entries indicate women carrying more than one diagnosis and are shown as a single row, which is why the subcategory counts in Table 1 of the main text exceed the number of women in some groups.

S2a. Chronic medical conditions predating the index pregnancy (37 women, 29.6%)

Diagnosis as recorded	n
Hypothyroidism	5
Migraine	3
Asthma	2
Epilepsy	2
Factor V Leiden, heterozygous	2
Familial Mediterranean fever	2
Hyperthyroidism	2
Allergic asthma	1
Brain tumour	1
Dysgerminoma (history of chemotherapy)	1
Grade 1 hepatic steatosis + thrombocytopenia + hypersplenism	1
HIV infection	1
Chronic hypertension	1
Chronic hypertension + factor V Leiden, heterozygous	1
Chronic hypertension + hypothyroidism	1
Hypothyroidism + asthma	1

Mitral regurgitation	1
Neurogenic bladder	1
Obesity	1
Restrictive lung disease + hypothyroidism + kyphoscoliosis	1
Systemic lupus erythematosus + Sjögren syndrome + immune thrombocytopenia	1
Supraventricular tachycardia	1
Thalassaemia minor	1
Varicose veins	1
Varicose veins + achalasia	1
Wolff–Parkinson–White syndrome	1
**Total**	**37**

S2b. Complications first diagnosed during the index pregnancy (42 women, 33.6%)

**Diagnosis as recorded**	**n**
Gestational diabetes mellitus	13
Hypothyroidism	11
Intrahepatic cholestasis of pregnancy	3
Hydronephrosis	3
Placenta praevia totalis	3
Preeclampsia	3
Gestational diabetes mellitus + hypothyroidism	2
Preterm prelabour rupture of membranes + chorioamnionitis	1
Gestational hypertension + gestational diabetes mellitus	1
Hydronephrosis + hypothyroidism	1
Hypothyroidism + placenta praevia totalis	1
**Total**	**42**

Hypothyroidism was recorded under two spellings in the source database (9 and 2 entries respectively); these have been merged. Thyroid disease appearing here was first diagnosed during the index pregnancy; women with pre-existing thyroid disease appear in S1a.

S2c. Indications for caesarean section (80 women)

Indication as recorded	n
Previous caesarean section	36
Cephalopelvic disproportion	13
Placenta praevia totalis	4
Breech presentation	4
Maternal disease	4
Failure to progress in labour	4
Fetal distress	4
Twin pregnancy	3
Placental abruption	2
Severe preeclampsia	1
Severe preeclampsia + previous caesarean section	1
Previous caesarean section + chorioamnionitis	1
Fetal anomaly	1
Fetal macrosomia	1
COVID-19 with deterioration in general condition	1
**Total**	**80**

S2d. COVID-19 vaccine products and doses

Product and number of doses	Before infection, n	After infection, n
BNT162b2, 1 dose	4	14
BNT162b2, 2 doses	4	28
BNT162b2, 3 doses	2	5
BNT162b2, 4 doses	0	3
CoronaVac, 1 dose	1	2
CoronaVac, 2 doses	1	2
CoronaVac, 3 doses	1	0
CoronaVac, 4 doses	0	1
BNT162b2 1 dose + CoronaVac 2 doses	0	1
BNT162b2 2 doses + CoronaVac 2 doses	0	1
BNT162b2 3 doses + CoronaVac 2 doses	0	1
CoronaVac 2 doses + BNT162b2 1 dose	0	1
**Any vaccination**	**13 (10.4%)**	**59 (47.2%)**
**No vaccination**	**112 (89.6%)**	**66 (52.8%)**

S2e. Pregnancies excluded because they did not end in a live birth (8 of 133)

Outcome	Gestational age at loss, weeks	Gestational age at positive PCR, weeks	Attribution
Pregnancy loss	7	7	Not attributable to SARS-CoV-2
Pregnancy loss	10	10	Not attributable to SARS-CoV-2

Pregnancy loss	11	11	Not attributable to SARS-CoV-2
Pregnancy loss	12	12	Not attributable to SARS-CoV-2
Pregnancy loss	16	14	Not attributable to SARS-CoV-2
Pregnancy loss	18	18	Not attributable to SARS-CoV-2
Neonatal death < 1 h	27	6	Lethal inherited fetal anomaly
Neonatal death < 1 h	39	8	Lethal inherited fetal anomaly

Four of the six pregnancy losses occurred in the first trimester and two in the second. In every case the loss was judged on clinical grounds not to be attributable to SARS-CoV-2 infection. Both neonatal deaths followed delivery of an infant with a lethal inherited anomaly and occurred within one hour of birth.
