# Supplementary material for: Maternal and Neonatal Outcomes in Pregnancies with PCR-Confirmed SARS-CoV-2 Infection Ending in Live Birth: A Single-Centre Turkish Cohort Benchmarked Against National Reference Data
Source: J Clin Med. 2026 Sep 11;15(18):7069. doi: 10.3390/jcm15187069 (PMC13607355; doi:10.3390/jcm15187069)
Supplement: Supplementary file 1 [file jcm-15-07069-s001.zip › Elci O Supplementary file S3 data dictionary.pdf]

Supplementary File S3

Analysis code, data dictionary and coding rules

Maternal and Neonatal Outcomes in Pregnancies with PCR-Confirmed SARS-CoV-2 Infection Ending in Live Birth: A Single-Centre Turkish Cohort Benchmarked Against National Reference Data

1. Purpose and scope

This document accompanies the analysis code supplied with the article. Together they allow a reader to inspect exactly how every value reported in Tables 1–5 and in Figure 1 was derived from the source clinical database, and to re-execute the analysis if the row-level data are obtained.

The row-level dataset itself is not distributed publicly. It consists of individual-level clinical records collected under an ethics approval that did not cover public data sharing, and the combination of rare diagnoses, gestational ages and outcomes within a single-centre cohort of this size carries a residual risk of re-identification. The anonymised dataset is available from the corresponding author on reasonable request, as stated in the Data Availability Statement.

2. Files provided

File	Contents
`Elci_analysis_code.py`	Python script that reproduces the participant flow, Tables 1–5 and the sensitivity analyses from the source workbook.
This document	Data dictionary for the source variables, definitions of every derived variable, and the coding and analysis rules applied.
Tables S1, S2	Diagnosis-level listings and the completed STROBE checklist (supplied separately).

3. Running the code

The script requires Python 3.10 or later with `pandas`, `numpy` and `scipy`. It takes the path to the source workbook as its only argument and writes a plain-text report to standard output:

```
python Elci_analysis_code.py DATA_COVID.xlsx > results.txt
```

The analyses reported in the manuscript were produced with Python 3 and SciPy; descriptive statistics were cross-checked against the IBM SPSS Statistics version 27.0 output generated for the original thesis analysis.

4. Source variables

The source workbook contains one row per pregnancy. Column headings are in Turkish, as recorded in the clinical database; the English gloss and the permitted values are given below. Free-text fields were entered by the clinical team and are reproduced verbatim in Supplementary Table S1.

Column (source)	English gloss	Type and permitted values
`YAŞ`	Maternal age	Integer, years.
`COVID (+) GH`	Gestational age at first positive PCR	Integer, completed weeks.
`COVID (+) TRİMESTER`	Trimester of first positive PCR	1, 2 or 3. See §6 for boundaries.
`GRAVİDA`	Gravidity	Integer, including the index pregnancy.
`PARİTE`	Parity	Integer, births before the index pregnancy.
`ABORTUS`	Previous abortions	Integer.
`ENFEKSİYON ÖNCESİ COVİD AŞISI`	COVID-19 vaccination before the infection	Free text, product and dose count, e.g. “2 DOZ BİONTECH”; “YOK” = none.

Column (source)	English gloss	Type and permitted values
`ENFEKSİYON SONRASIİ COVİD AŞISI`	COVID-19 vaccination after the infection	As above. The heading contains a typographical error in the source file and is used as-is.
`KRONİK HASTALIKLARI`	Chronic medical conditions	Free text; multiple diagnoses separated by “+”; “YOK” = none. Conditions predating the index pregnancy only.
`GEBELİK SÜRECİNDE GELİŞEN MATERNAL KOMORBİDİTE`	Complications of the index pregnancy	Free text, as above. Conditions first diagnosed during the index pregnancy only.
`DOĞUM HAFTASI`	Gestational age at delivery	Integer, completed weeks. For excluded pregnancies this records the gestational age at loss.
`DOĞUM ŞEKLİ`	Mode of delivery	“C/S” = caesarean section; “NSD” = vaginal delivery.
`C/S İSE ENDİKASYON`	Indication for caesarean section	Free text; two indications may be combined with “+”. Blank for vaginal deliveries.
`ÖLÜ DOĞUM/ABORT`	Pregnancy loss	“YOK” = live birth; “ABORTUS” = pregnancy loss before 20 weeks; “POSTNATAL 1. SAAT EXİTUS” = neonatal death within one hour of birth.
`PEROPERATİF KOMPLİKASYON GELİŞİMİ`	Intraoperative or immediate postpartum complication	Free text; “YOK” = none.
`DOĞUMDA ANNENİN HOSPİTALİZASYON SÜRESİ`	Maternal length of hospital stay	Free text, e.g. “3 GÜN” (3 days).
`ANNEDE YBÜ İHTİYACI`	Maternal intensive care	Free text length of stay, e.g. “8 GÜN”; “YOK” = not required.
`COVID İÇİN VERİLEN TEDAVİ`	Pharmacological treatment for COVID-19	Free text, e.g. “CLEXANE”; “YOK” = none. “CLEXANE” denotes enoxaparin, a low-molecular-weight heparin.
`YENİDOĞAN YBÜ İHTİYACI`	Neonatal intensive care	Free text length of stay, e.g. “9 GÜN”; “YOK” = not required. For twins, both neonates are described, e.g. “BEBEK1 YOK BEBEK2 10 GÜN”.
`1. DK APGAR`	Apgar score at one minute	Integer 0–10. For twins, both scores, e.g. “5 VE 5” (5 and 5). “ENTÜBE” = intubated, no score recorded.
`5. DK APGAR`	Apgar score at five minutes	As above.

Turkish terms appearing in free-text fields: YOK = none/absent; GÜN = day(s); DOZ = dose(s); VE = and; BEBEK = infant; MÜKERRER = repeat (caesarean); EXİTUS = death.

5. Derived variables

Variable	Definition
`preterm`	Gestational age at delivery < 37 completed weeks.
`caesarean`	Mode of delivery recorded as C/S.
`twin`	Caesarean indication recorded as twin pregnancy. Three such pregnancies; the cohort of 125 pregnancies therefore comprises 128 neonates.
`apgar1_low`	Apgar score at one minute. For twin pregnancies, the lower of the two recorded scores.
`apgar5_low`	Apgar score at five minutes, defined as above.
`mat_los_d`	Maternal length of stay in days, parsed from the free-text field.
`stay_gt3`	Maternal length of stay strictly greater than three days.
`nicu_days`	Neonatal intensive care length of stay in days. Where both twins are described, the longer stay is taken.
`nicu_any`	Any neonatal intensive care admission, i.e. a length of stay was recorded.
`mat_icu_any`	Any maternal intensive care admission, defined as above.
`chronic_any`	Any chronic medical condition recorded.
`comorb_any`	Any complication of the index pregnancy recorded.
`vax_pre_any`	Any COVID-19 vaccine dose recorded before the infection.
`vax_post_any`	Any COVID-19 vaccine dose recorded after the infection.
`treated`	Any pharmacological treatment directed at COVID-19 recorded.
`perop_any`	Any intraoperative or immediate postpartum complication recorded.

6. Coding and analysis rules

The following decisions were taken before the analysis and are implemented in the code. They are listed here because several of them affect the reported values and would otherwise not be recoverable from the manuscript alone.

Cohort definition. The source file contains the 133 pregnancies for which clinical records could be retrieved; the 18 women whose records could not be retrieved are not present in it. Eight pregnancies that did not end in a live birth were excluded by the code: six pregnancy losses before 20 weeks (gestational ages 7, 10, 11, 12, 16 and 18 weeks) and two neonatal deaths within one hour of birth (at 27 and 39 weeks). The analysis cohort is therefore 125 pregnancies.

Trimester boundaries. First trimester, up to 13 weeks and 6 days; second, 14 weeks and 0 days to 27 weeks and 6 days; third, 28 weeks and 0 days onwards. The trimester was recorded in the source database and was not re-derived from the gestational age.

Chronic condition versus pregnancy complication. A diagnosis was classified as a chronic medical condition if it predated the index pregnancy and as a complication of the index pregnancy if it was first made during it. The two are recorded in separate columns and the categories are mutually exclusive. Hypothyroidism occurs in both, and was assigned on this basis.

Twin pregnancies. Analyses are at the level of the pregnancy, not the neonate. For the three twin pregnancies the lower of the two Apgar scores was used, so that a pregnancy is characterised by its worse outcome, and a pregnancy was counted as requiring neonatal intensive care if either twin was admitted. Where one twin was intubated and no Apgar score was recorded, the recorded co-twin value was used.

Prolonged maternal stay. Defined a priori as a stay of more than three days, because the routine post-caesarean stay at the institution was extended from 48 to 72 hours during the pandemic.

Data-entry correction. Two rows of the vaccination-before-infection column contain “YIOK”, a typographical error for “YOK” (none). These are normalised to “YOK” by the code. No other values were altered. Leading and trailing

whitespace and inconsistent capitalisation in free-text fields are normalised before tabulation, which merges entries such as "CLEXANE" and "CLEXANE ".

Statistical tests. Distributions were examined with the Kolmogorov–Smirnov test; because several continuous variables were non-normally distributed, all continuous between-group comparisons use the Mann–Whitney U test. Categorical comparisons use the chi-square test without continuity correction, or the Fisher exact test where any expected cell count falls below five; the test actually applied is printed alongside each result. Cohort proportions are compared with national reference values by one-sample exact binomial tests with Clopper–Pearson confidence intervals. All tests are two-sided. No adjustment was made for multiple comparisons.

National reference values. Caesarean share of live births 61.2% and maternal mortality ratio 11.5 per 100,000 live births (Health Statistics Yearbook 2024); neonatal mortality rate 5.6 per 1000; preterm birth rate derived as 121,067 preterm births divided by 937,559 live births for 2024. These are set as constants at the top of the script and can be varied. The script also prints the sensitivity analysis across the plausible range of published values that is reported in Section 3.6 of the manuscript.

Early neonatal death denominator. The comparison of early neonatal death against the national neonatal mortality rate uses all 127 live births among the 133 pregnancies with retrievable records, that is the analysis cohort plus the two neonates who died within one hour of birth, and not the 125-pregnancy cohort.

7. Reproducibility check

Running the script on the source workbook reproduces every value in Tables 1–5 of the manuscript, the counts in Figure 1, and the sensitivity analysis. The printed report is organised under the same table headings as the manuscript to make the correspondence explicit. Verbatim tallies of the free-text diagnosis, vaccine, treatment and caesarean-indication fields are printed under Tables 1 and 2 and correspond to Supplementary Table S1.
