# Supplementary material for: Maternal and Neonatal Outcomes in Pregnancies with PCR-Confirmed SARS-CoV-2 Infection Ending in Live Birth: A Single-Centre Turkish Cohort Benchmarked Against National Reference Data
Source: J Clin Med. 2026 Sep 11;15(18):7069. doi: 10.3390/jcm15187069 (PMC13607355; doi:10.3390/jcm15187069)
Supplement: Supplementary file 1 [file jcm-15-07069-s001.zip › Elci Supplementary Tables S4-S7.pdf]

Supplementary Tables S4–S7

Added at first revision in response to reviewer comments

Maternal and Neonatal Outcomes in Pregnancies with PCR-Confirmed SARS-CoV-2 Infection Ending in Live Birth: A Single-Centre Turkish Cohort Benchmarked Against National Reference Data

Table S4. Effect sizes for the comparisons in Table 3 (neonatal intensive care unit admission)

Variable	Risk difference	95% CI	Odds ratio (95% CI)
**Preterm birth < 37 weeks**	**+47.1%**	**+22.2 to +72.0**	**12.73 (3.90–41.53)**
**Treated for COVID-19**	**+37.6%**	**+13.2 to +61.9**	**4.85 (1.56–15.06)**
Maternal stay > 3 days	+21.6%	−4.4 to +47.5	2.52 (0.87–7.30)
Caesarean delivery	+19.8%	−1.4 to +41.0	2.72 (0.73–10.10)
Complication of current pregnancy	+18.8%	−7.2 to +44.8	2.21 (0.76–6.37)
Vaccinated after infection	+10.4%	−15.7 to +36.4	1.52 (0.53–4.37)
Chronic medical condition	−12.4%	−33.5 to +8.6	0.51 (0.14–1.90)

Continuous variables, Hodges–Lehmann median difference with bootstrap 95% CI (2000 resamples): gestational age at delivery −3.0 weeks (−5.0 to 0.0); Apgar score at 1 min −1.0 (−2.0 to 0.0); Apgar score at 5 min −1.0 (−1.0 to 0.0). Risk differences are for the NICU group relative to the non-NICU group; a positive value indicates a higher proportion among neonates admitted to intensive care. Odds ratios use the Haldane correction where a zero cell is present. Rows in bold reach conventional significance in Table 3; no adjustment was made for multiple comparisons.

Table S5. Effect sizes for the comparisons in Table 4 (maternal hospital stay > 3 days)

Variable	Risk difference	95% CI	Odds ratio (95% CI)
**Caesarean delivery**	**+41.1%**	**+27.7 to +54.6**	**11.45 (3.28–40.04)**
**Treated for COVID-19**	**+33.4%**	**+15.4 to +51.4**	**4.18 (1.88–9.32)**
**Complication of current pregnancy**	**+33.2%**	**+15.2 to +51.1**	**4.27 (1.91–9.57)**
**Preterm birth < 37 weeks**	**+22.6%**	**+7.0 to +38.2**	**5.02 (1.79–14.04)**
Vaccinated after infection	+17.1%	−1.5 to +35.7	2.00 (0.93–4.31)
NICU admission	+11.2%	−2.9 to +25.3	2.52 (0.87–7.30)
Chronic medical condition	+5.4%	−12.1 to +23.0	1.29 (0.57–2.92)

Continuous variables, Hodges–Lehmann median difference with bootstrap 95% CI: gestational age at delivery −1.0 week (−2.0 to 0.0); Apgar score at 1 min −1.0 (−2.0 to 0.0); Apgar score at 5 min −1.0 (−1.0 to 0.0). Risk differences are for the group staying more than three days relative to the group staying three days or fewer. Rows in bold reach conventional significance in Table 4; no adjustment was made for multiple comparisons.

Table S6. Sensitivity analysis including all 133 pregnancies with retrievable records

Outcome	Result	National rate	p
Preterm birth < 37 weeks [a]	20/127 (15.7%), 95% CI 9.9–23.3	12.9%	0.35
Caesarean delivery [a]	81/127 (63.8%), 95% CI 54.8–72.1	61.2%	0.59
Early neonatal death	2/127 (1.6%), 95% CI 0.2–5.6	5.6 / 1000	0.16
Pregnancy not ending in live birth	8/133 (6.0%)	—	—
Composite adverse outcome [b]	26/133 (19.5%)	—	—
Preterm birth by NICU admission	9/16 vs. 17/117	—	< 0.001
Preterm birth by maternal stay > 3 days	12/39 vs. 14/94	—	0.036

[a] Referred to the 127 births at or beyond 20 weeks of gestation. The six pregnancy losses occurring at 7, 10, 11, 12, 16 and 18 weeks are not births and cannot be classified as preterm or by mode of delivery; including them in the numerator of a preterm birth rate would be a category error, and they are therefore counted only in the two rows that treat non-live birth as an outcome in its own right. [b] Neonatal intensive care admission, maternal intensive care admission, or a pregnancy not ending in live birth. No conclusion of the primary analysis is altered by including the excluded pregnancies.

Table S7. Exploratory multivariable logistic regression

Outcome and covariate	Adjusted odds ratio	95% CI	p
**Neonatal intensive care admission (16 events, n = 125)**			
Preterm birth < 37 weeks	14.20	3.60–56.05	< 0.001
Complication of current pregnancy	0.79	0.20–3.12	0.735
Maternal age, per year	1.01	0.90–1.12	0.891
**Maternal hospital stay > 3 days (39 events, n = 125)**			
Preterm birth < 37 weeks	3.09	1.01–9.41	0.047
Complication of current pregnancy	3.16	1.34–7.49	0.009
Maternal age, per year	1.04	0.97–1.12	0.283

Models were fitted by maximum likelihood. Covariates were limited to three because of the small number of events: with 16 neonatal intensive care admissions the number of events per variable is approximately five, well below the conventional requirement of ten, and the confidence intervals are correspondingly wide. Mode of delivery was deliberately not entered in the model for prolonged maternal stay, since the length-of-stay threshold is itself defined by the routine post-caesarean discharge interval and the two variables are not independent. These estimates are exploratory and are presented to show that the univariable associations are not obviously accounted for by the covariates entered; they should not be read as adjusted risk estimates.
